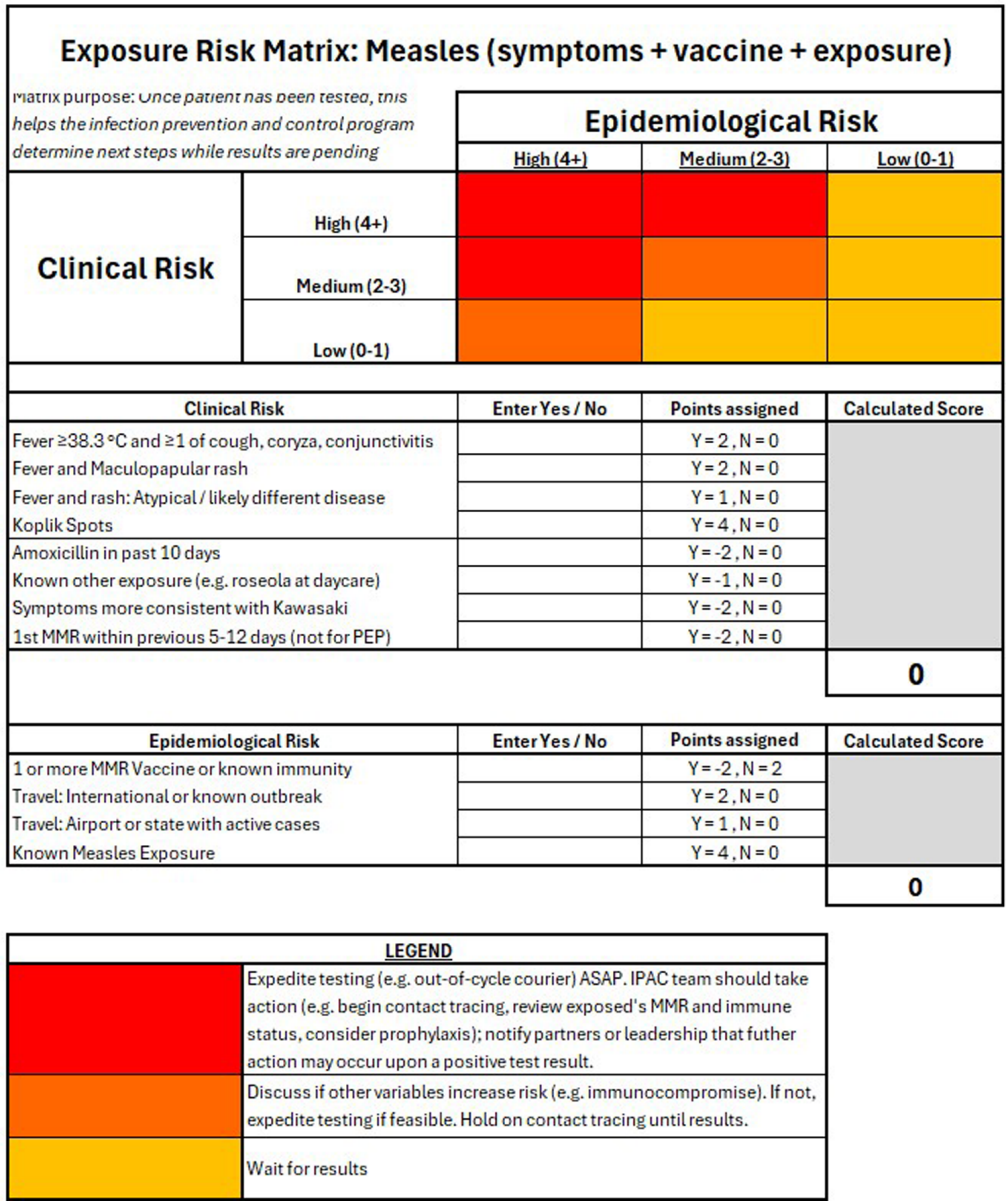# 54 From Benchmark to Best Practice: Reducing Blood Culture Contamination in Sepsis Care

**DOI:** 10.1017/ash.2026.10488

**Published:** 2026-06-23

**Authors:** Brad Krier, Jean Barth, W. Charles Huskins

**Affiliations:** 1 Mayo Clinic Health System- Mankato; 2 Mayo Clinic

## Abstract

**Background:** A measles case requires immediate action to identify and administer prophylaxis to exposed individuals in healthcare settings. Infection preventionists (IPs) often balance proactive contact tracing with waiting for a positive test result. Objective: To develop a tool that enables IPs to stratify suspect measles cases by risk level, thereby supporting the decision to prioritize or defer active intervention while awaiting test results. **Methods:** In spring 2025, we developed a preliminary matrix and used 19 additional suspect patients to further refine the scoring of the tool. In August 2025, we deployed a finalized tool. The two-dimensional risk matrix incorporated both epidemiological risk factors and clinical risk factors, each scored numerically based on yes or no questions. The sum of both categories was used to assign a final risk level that determined the recommendations for IPs (Figure 1). The study population included patients tested for measles during the implementation period. Descriptive statistics were used to summarize the distribution of risk categories and outcomes. Among the whole cohort, Firth’s logistic regression was used to evaluate the association between clinical or epidemiological risk score and measles diagnosis. Among the production cohort, sensitivity, specificity, positive predictive value (PPV) and negative predictive value (NPV) were calculated to evaluate the diagnostic performance. **Results:** Since implementation, the matrix was applied to 56 patients tested for measles. Of these, 10.7% (n=6) were categorized as high risk, 32.1% (n=18) as medium, and 57.1% (n=32) as low. Four of the 56 patients were positive for measles, 3 were categorized as high-risk and 1 as medium risk. Each one-unit increase in epidemiological risk was associated with a 2 folds increase in the odds of a positive measles diagnosis (odds ratio = 2.0; 95% CI, 1.3–3.6). The low-risk category had an NPV of 1, indicating that all low?risk patients tested negative, thus supporting safe workload reduction by deprioritizing these cases. **Conclusion:** The Measles Exposure Response Risk Matrix effectively stratified cases and supported IP prioritization efforts. Low-risk groups required no action, expedited testing was reasonable for medium-risk cases, and proactive actions were reserved for high-risk cases. Clinical and epidemiologic risk scores progressed from arbitrary assignment to validated metrics, confirmed by statistical analysis. The flexible design permits adaptation to other infectious diseases, including avian influenza and mpox. With rising measles incidence, tools like this matrix will be essential to support rapid, evidence-based actions by infection prevention teams.

**Figure:**